# Mineralocorticoid receptor antagonism attenuates arteriovenous fistula stenosis by modulating the phenotype of vascular smooth muscle cells

**DOI:** 10.1093/ndt/gfae247

**Published:** 2024-11-07

**Authors:** Yamin Liu, Bohan Chen, Kai Chen, Yufei Wang, Chunyu Zhou, Xianhui Liang, Kai Wang, Pei Wang

**Affiliations:** Blood Purification Center, Department of Nephrology, the First Affiliated Hospital of Zhengzhou University, Zhengzhou, Henan, China; Key Laboratory of Bioactive Materials, Ministry of Education, College of Life Science, Nankai University, Tianjin, China; Blood Purification Center, Department of Nephrology, the First Affiliated Hospital of Zhengzhou University, Zhengzhou, Henan, China; Research Institute of Nephrology, Zhengzhou University, Zhengzhou, China; Department of Nephrology, Kaifeng People's Hospital, Kaifeng, China; Blood Purification Center, Department of Nephrology, the First Affiliated Hospital of Zhengzhou University, Zhengzhou, Henan, China; Blood Purification Center, Department of Nephrology, the First Affiliated Hospital of Zhengzhou University, Zhengzhou, Henan, China; Blood Purification Center, Department of Nephrology, the First Affiliated Hospital of Zhengzhou University, Zhengzhou, Henan, China; Research Institute of Nephrology, Zhengzhou University, Zhengzhou, China; Blood Purification Center, Department of Nephrology, the First Affiliated Hospital of Zhengzhou University, Zhengzhou, Henan, China; Key Laboratory of Bioactive Materials, Ministry of Education, College of Life Science, Nankai University, Tianjin, China; Blood Purification Center, Department of Nephrology, the First Affiliated Hospital of Zhengzhou University, Zhengzhou, Henan, China; Research Institute of Nephrology, Zhengzhou University, Zhengzhou, China

**Keywords:** arteriovenous fistula, finerenone, mineralocorticoid receptor, phenotypic switching, vascular smooth muscle cells

## Abstract

**Background:**

Fistula stenosis is a primary contributor to arteriovenous fistula (AVF) failure in maintenance hemodialysis patients. Emerging data indicated excessive fibrotic remodeling was the primarily contributor to fistula stenosis during AVF remodeling. The mineralocorticoid receptor (MR) has been implicated in vascular remodeling across various cardiovascular pathologies. However, its role in AVF remodeling, particularly concerning fibrotic remodeling, remains elusive.

**Methods:**

MR expression and the phenotypes of vascular smooth muscle cells (VSMC) were assessed in dysfunctional AVF. The effects of MR on VSMC phenotypic switching were examined *in vitro*, and the protective effects of MR antagonists on AVF outcome were evaluated in a rat AVF model.

**Results:**

Dysfunctional fistula exhibited significant medial fibrosis and extracellular matrix deposition, alongside markedly increased MR activity. In the dysfunctional fistula vessels, VSMC displayed reduced expression of the contractile marker SMMHC and features characteristic of a synthetic phenotype, including increased osteopontin expression and heightened proliferation. *In vitro* studies with cultured VSMC revealed that MR overactivity induced by aldosterone led to phenotypic switching from contractile to synthetic state, concomitant with EGFR-ERK1/2 pathway overactivation. These effects were largely abolished by the MR antagonist finerenone. Knockdown of EGFR expression abrogated ERK1/2 phosphorylation and inhibited the VSMC phenotypic switching. Conversely, ectopic overexpression of EGFR in VSMC diminished the protective effect of finerenone. In rat AVF models, pharmacologic targeting of MR with finerenone significantly improved AVF outcomes, characterized by increased luminal diameters and flow volume, reduced medial fibrosis, and inhibited VSMC phenotypic switching. These beneficial outcomes were likely attributable to a restrained activity of the EGFR-ERK1/2 pathway in VSMC.

**Conclusions:**

Our study demonstrated that therapeutic targeting of MR may improve AVF outcome by modulating VSMC phenotypic switching. These findings offer promising avenues for further clinical investigations aimed at optimizing AVF outcomes in the hemodialysis population.

## Graphical Abstract

**Graphical Abstract:**
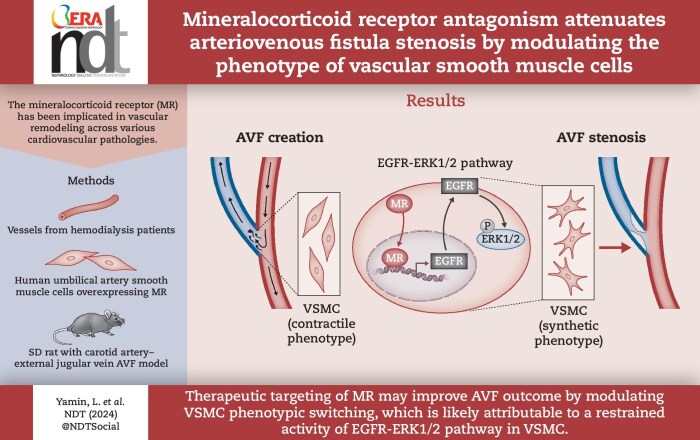

KEY LEARNING POINTS **What was known:**

- Arteriovenous fistula (AVF) failure remains a significant clinical challenge in patients undergoing maintenance hemodialysis, primarily attributed to fistula stenosis. Recent studies have highlighted the potential involvement of the mineralocorticoid receptor (MR) in vascular remodeling, prompting us to investigate its role in AVF dysfunction.

**This study adds:**

- Through *in vitro* studies with cultured vascular smooth muscle cells (VSMC), we delineated the mechanism by which MR overactivity mediated by the EGFR-ERK1/2 pathway. In rat AVF models, pharmacological targeting of MR with finerenone significantly improved AVF outcomes, characterized by increased luminal diameters and flow volume, reduced medial fibrosis, and inhibited VSMC phenotypic switching.

**Potential impact:**

- Our study elucidated the molecular mechanisms underlying AVF dysfunction and providing translational insights into potential therapeutic interventions. By targeting MR, we offer a promising avenue for mitigating AVF failure and improving outcomes in hemodialysis patients.

## INTRODUCTION

Hemodialysis stands as the predominant modality of renal replacement therapy for patients with end-stage renal disease (ESRD). Among the available vascular access options, arteriovenous fistula (AVF) represents the optimal choice in terms of the longest patency duration, the lowest risk of infections, and the fewest complications when compared to arteriovenous graft and central venous catheters [1]. Nevertheless, recent findings underscore that the patency of AVF may be compromised by venous

stenosis, stemming from excessive fibrotic remodeling within the venous vasculature [2, 3]. Despite efforts utilizing interventions such as balloon angioplasty [4] and antiplatelet agents [5, 6], the preservation of AVF patency has remained a daunting challenge. Hence, a novel therapeutic agent targeting venous fibrosis of AVF was urgently needed.

Mineralocorticoid receptor (MR), a nuclear transcription factor belonging to the steroid receptors family, exhibits diverse pathophysiological roles within the cardiovascular system, beyond its classical function in renal sodium regulation [7, 8]. A myriad of clinical studies have implicated that MR is involved in the pathogenesis of various cardiovascular disorders, including heart failure, hypertension, and myocardial infarction [9]. MR antagonists have salutary effects on mitigating cardiac remodeling and significantly reducing morbidity and mortality associated with cardiovascular diseases [10]. Consistently, it has been observed that patients with primary aldosteronism, characterized by elevated levels of the MR agonist aldosterone, manifest aberrant vascular function [11]. Experimental studies further elucidate the detrimental consequences of MR activation in response to vascular injury, exacerbating vascular dysfunction encompassing constriction and fibrosis [12]. Conversely, inhibition of MR by gene knockout or antagonists such as spironolactone or finerenone demonstrates a capacity to ameliorate vascular fibrosis in vein graft models or vascular injury models [13–15].

As such, MR plays an instrumental role in cardiovascular remodeling. However, the specific role of MR in venous fibrosis of AVF, which is implicated in the process of vascular remodeling, remains poorly understood. Considering the beneficial cardiovascular effects of MR antagonists, in this study, we explored the involvement of MR in AVF venous fibrosis and whether MR antagonists, finerenone, could confer protective effects on AVF remodeling *in vitro* in cultured vascular smooth muscle cells (VSMC), *in vivo* in a rat AVF model.

## MATERIALS AND METHODS

### Human vessel samples

All vessel samples were collected at the First Affiliated Hospital of Zhengzhou University. Dysfunctional AVF veins were captured at the time of surgical revision. Cephalic vein, which served as a control of vein, was collected at the time of AVF creation.

### Cell culture and transfection

Human umbilical artery VSMC was obtained from Aoyinbio Co. (Shanghai, China) and was grown in Dulbecco's modified Eagle's medium/F12 containing 10% FBS at 37 °C [16]. Cells were transfected with lentivirus coding for human NR3C2 cDNA, human EGFR cDNA, or siRNA-EGFR (GENECHEM, China) according to the manufacturer's instructions. After stable transfection, cells were treated with aldosterone (100 nM, Sigma-Aldrich, Germany) or finerenone (40 nM, Sigma-Aldrich) treatment for 24 h. After the indicated treatment, cells were processed for further examination.

### Rat AVF model

Sprague Dawley (SD) rats, male, weighing 300∼350 g were purchased from Beijing Vital River Laboratory Animal Technology Co., Ltd (Beijing, China). The rats were fed in the laminar laboratory of the Experimental Animal Center of Nankai University (Tianjin, China), with a feeding temperature of 25 °C and a 12/12 h light/dark cycle. All animal experiments were approved by the Animal Experiments Ethical Committee of Nankai University and performed with the NIH Guide for Care and Use of Laboratory Animals.

A 20 G disposable venous indwelling needle catheter (TOGO MEDIKIT CO., LTD., Japan), ∼4 mm segments with a ∼2 mm handle was cut to as a cuff.

Fifteen SD rats were randomly divided into three experimental groups (Sham, vehicle, and finerenone group). Rats were anesthetized through intraperitoneal administration of sodium pentobarbital (60 mg/kg). After the neck was shaved and sterilely prepared, the rats were fixed in a supine position and the neck extended. In the vehicle and finerenone group, the operation was performed under a dissecting microscope. Through a midline skin incision of the neck, the common carotid artery and ipsilateral external jugular vein were dissected and exposed. All branches of the jugular vein were ligated with a 8–0 suture, a ∼1 cm vein bridge length was dissociated, and the lumen was flushed with saline (containing heparin sodium 50 U/ml). The common carotid artery was ligated just below the carotid bifurcation with two 8–0 sutures, and transected between them. The vessel wall at the broken end of the artery was passed through a cuff; with gentle pulling, and using an arterial clamp to hold the artery and the handle of the cuff, then the artery was flipped outwards to the cuff and tied with 8-0 suture. The flipped artery was inserted into the reversed vein bridge and tied with 8–0 sutures twice. The handle was cut off gently. The operative field and anastomosis were irrigated with a saline solution containing gentamycin sulfate of 160 U/ml. The skin was then closed with continuous stitches using a 3–0 suture. In the sham group, the same incision position was adopted, and following the freeing of the left submandibular gland and left sternocleidomastoid muscle, the left external jugular vein and the left carotid artery were separated by a noninvasive technique, and the incision was sutured. The finerenone group was intragastric with finerenone (10 mg/kg/d) for 21 days, which was dissolved in a solvent of 40% macrogol (15)-hydroxystearate (Solutol1, Cat. 42 966, Sigma-Aldrich) and 10% ethanol. The vehicle group was intragastric with solvent.

### Ultrasonography

Three weeks after surgery, the rats were anesthetized and imaged with the Vevo 2100 system (VisualSonics, Canada). B-mode and color Doppler were used to assess the luminal diameter and blood flow rate of the AVFs. A 21-MHz B mode probe was used to obtain measurements of the outflow vein luminal diameter, 2 mm from the distal end of the cuff. A 16-MHz Doppler probe was used to measure the blood flow rate at arteries, 5 mm from the proximal end of the cuff.

### Vessel histology assessment immunohistochemistry staining

Vessel specimens underwent formalin fixing, paraffin embedding, and 5-μm-thick sections preparation. Sections were processed for Masson's trichrome staining (Solarbio Life Science, China) by routine procedures or subjected to primary antibodies against fibronectin and collagen I followed by secondary antibodies and DAB kit (Servicebio, China). The micrographs were captured by using the Nikon upright microscope (Nikon Instruments). Computerized morphometry of immunohistochemical staining was analyzed by using Image-Pro Plus [17] as described previously.

### Immunofluorescent staining

Fixed cell and vessel sections were fixed with 4% paraformaldehyde, permeabilized by Triton X-100, and then stained with primary antibodies against MR, α-SMA, SMMHC, OPN, ki67, and calponin, followed by Alexa Flour conjugated secondary antibody. Finally, fixed cells were counterstained with propidium iodide or DAPI (Vector Laboratories, USA). The fluorescence micrographs were taken by a fluorescent microscope (Nikon Instruments).

### Western blot

Cultured cells were lysed and vessel tissues were homogenized in a radioimmunoprecipitation assay buffer containing protease inhibitors and PMSF. As described before [18], protein lysate was subjected to gel electrophoresis, protein transfer, blocking and primary antibody incubation with MR (Abcam, OR, USA), calponin, osteopontin (OPN), EGFR, p-ERK1/2, ERK1/2 (Proteintech, China), β-actin and GAPDH (Servicebio). The integrated density of the immunoblot bands was analyzed by using ImageJ software, version 1.52a (National Institutes of Health, Bethesda, MD, USA).

### Cell migration assay

30 min after different treatments, the confluent VSMC monolayer was scraped with a 10 μL pipette. Phase-contrast images of VSMC were captured at 0 h and 12 h. The migration area was quantified and expressed as the percentage of cell migration area.

### Statistics

The data are presented as mean ± SD. One-way ANOVAs followed by Tukey's tests were conducted to compare data from multiple groups. Two-tailed, unpaired Student's *t* tests were applied to compare data from two groups. The statistical analyses were conducted by using GraphPad Prism 8.0. A statistical significance was defined as *P* < 0.05.

## RESULTS

### MR overactivation was accompanied by VSMC phenotypic switching in dysfunctional AVF

**Figure 1:**
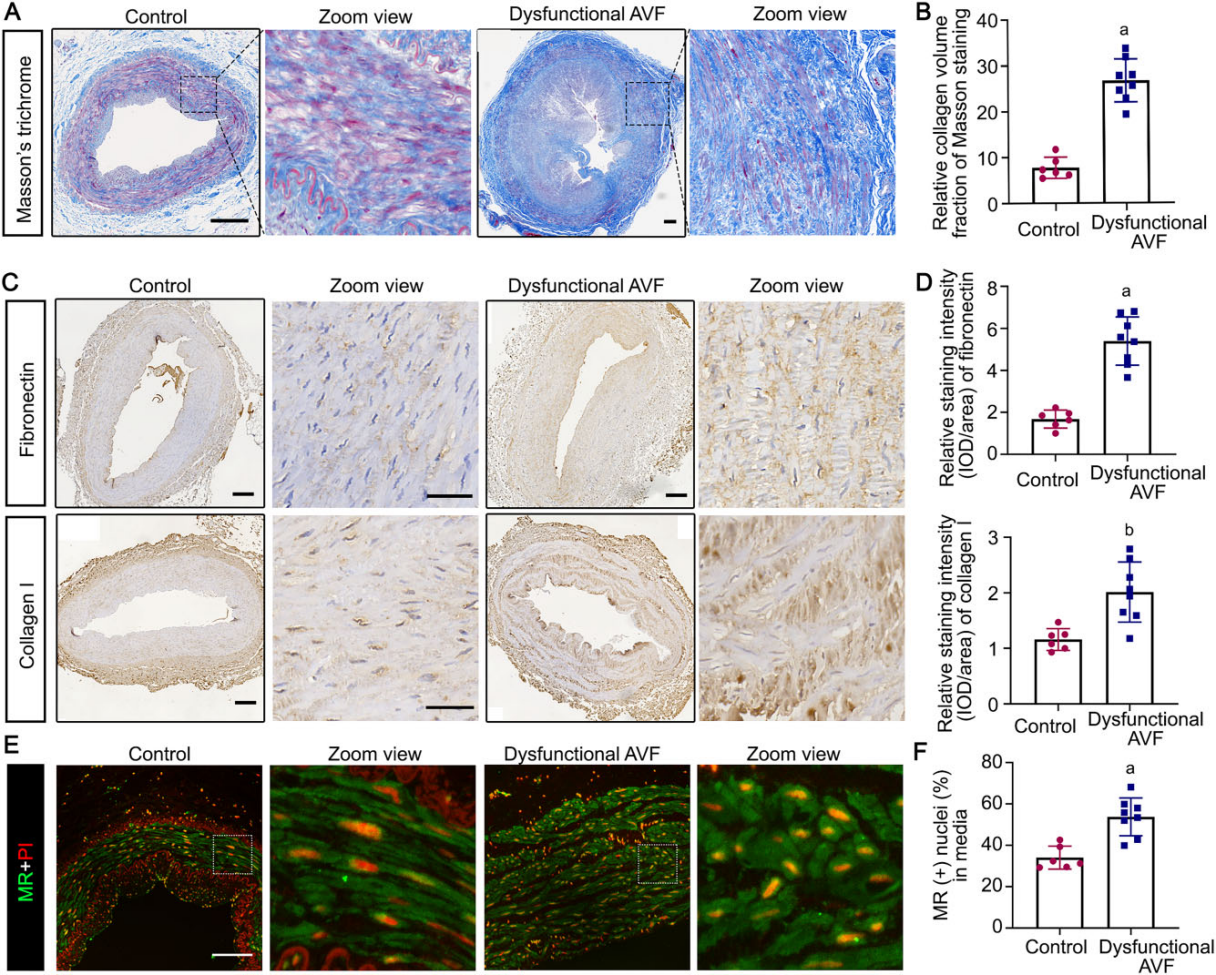
Mineralocorticoid receptor (MR) was overactivated in dysfunctional arteriovenous fistula (AVF). Dysfunctional AVF veins were captured at the time of revision surgery. The cephalic vein, which served as a control of vein, was collected before anastomosis of AVF creation. (**A**) Vascular sections were subjected to Masson's trichrome staining and representative images and zoom views are shown. Scale bar = 200 µm. (**B**) Quantification of collagen volume fraction of Masson's trichrome staining. ^a^*P* < 0.0001 versus control (n = 6 or 8). (**C**) Vascular sections were subjected to immunohistochemistry staining of fibronection or collagen I; representative images and zoom views are shown. Scale bar = 200 µm. (**D**) Quantification of fibronectin or collagen I staining intensity by morphometric analyses expressed as relative levels normalized to the control. ^a^*P* < 0.0001 versus control, ^b^*P* = 0.0033 versus control (n = 6 or 8). (**E**) Vascular sections were subjected to immunostaining of MR and counterstained with propidium iodide (PI). Scale bar = 50 µm. (**F**) Absolute counting of the number of MR-positive nuclei, expressed as percentage of the total number of cells per high-power field. ^a^*P* = 0.0006 versus control (n = 6 or 8).

The venous sample of dysfunctional AVF were collected at the time of revision surgery. Consecutive vascular sections were processed for Masson's trichrome staining or immunohistochemistry staining of fibronectin, collagen I, or MR. Shown in Fig. 1A, vascular medial fibrosis was noted in dysfunctional AVF, as shown by Masson's trichrome staining and semi-quantitative morphometric analysis of collagen volume fraction (Fig. 1B). Moreover, the deposition of extracellular matrix (ECM) like fibronectin and collagen I in the media of dysfunctional fistula were significantly increased, as shown by immunohistochemistry staining and computerized morphometric analysis (Fig. 1C&D). In parallel, MR was overactivated in the venous vessel of dysfunctional fistula, marked by increased nuclear accumulation of MR, as compared to the naive vessel (Fig. 1E&F).

**Figure 2:**
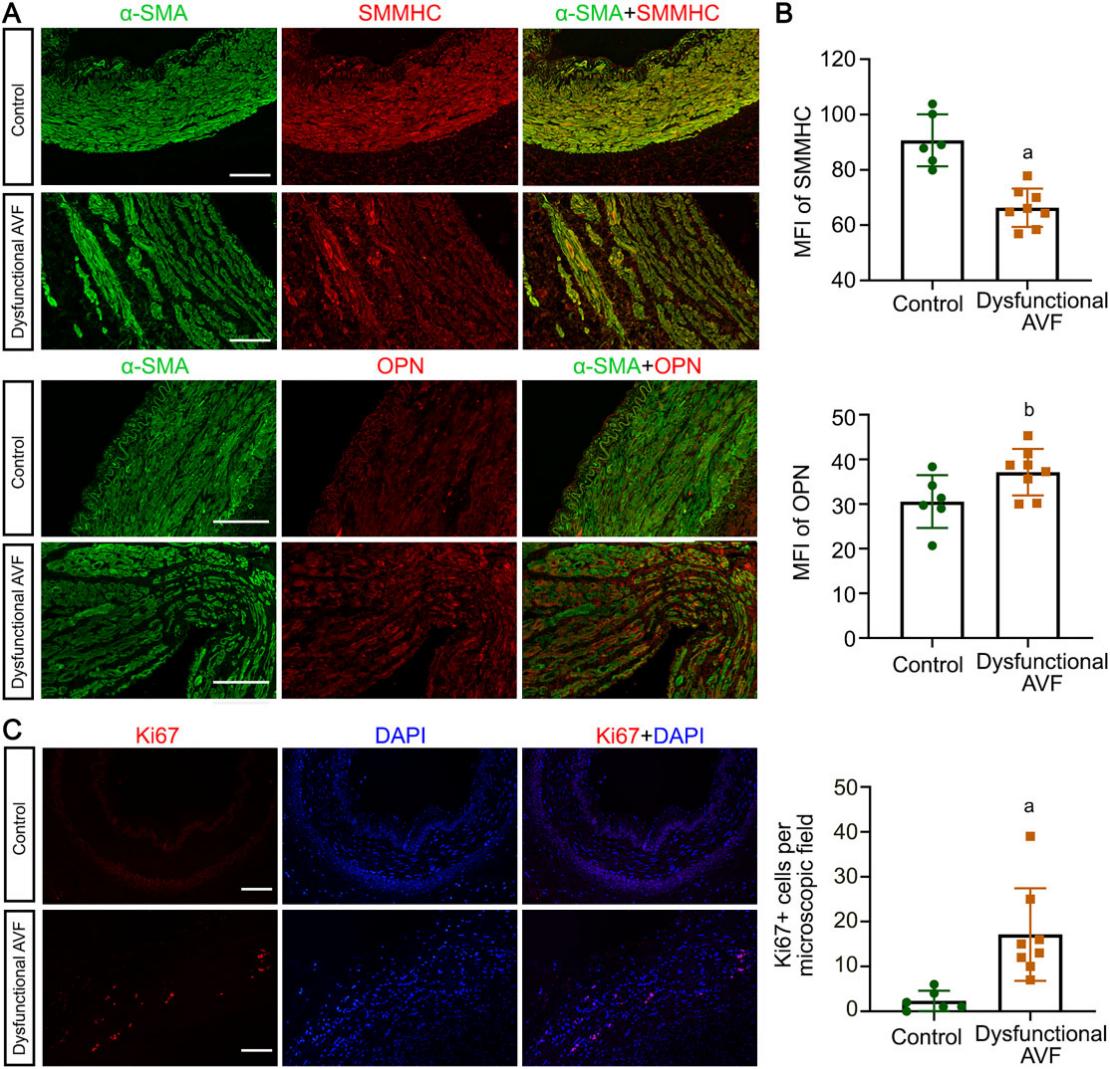
The phenotype of VSMC was switched in dysfunctional arteriovenous fistula (AVF). (**A**) Vascular sections of dysfunctional AVF were subjected to co-immunofluorescent staining of smooth muscle α-actin (α-SMA) with smooth muscle myosin heavy chain (SMMHC) or α-SMA with osteopontin (OPN). Representative micrographs are shown. Scale bar = 50 µm. (**B**) Quantification of mean fluorescence intensity (MFI) of SMMHC and OPN. ^a^*P* = 0.0001 versus control, ^b^*P* = 0.0469 versus control (n = 6 or 8). (**C**) Vascular sections were subjected to immunostaining of ki67 and quantification of ki67-positive cells per microscopic field. Scale bar = 50 µm. ^a^*P* = 0.0050 versus control (n = 6 or 8).

The phenotypic plasticity of VSMC plays a crucial role in vascular remodeling. Under normal physiological conditions, VSMC exhibits a differentiated, quiescent, contractile phenotype. However, in response to various stimuli, VSMC transition to a proliferative and synthetic phenotype. This phenotypic switching of VSMC has been well documented in the context of atherosclerosis. Nevertheless, its involvement in AVF vascular remodeling has yet to be elucidated. Shown in Fig. 2, the phenotypic switching of VSMC from contractile types to synthetic types in dysfunctional AVF was observed, evidenced by diminished expression of contractile VSMC markers, such as smooth muscle myosin heavy chain 11 (SM-MHC, also known as MYH11), coupled with increased expression of the synthetic VSMC marker OPN and increased ki67 positive cells, as shown by immunofluorescent staining (Fig. 2A&C) or computerized morphometric analysis (Fig. 2B).

### MR modulates VSMC phenotypic switching involving the EGFR-ERK1/2 pathway

**Figure 3:**
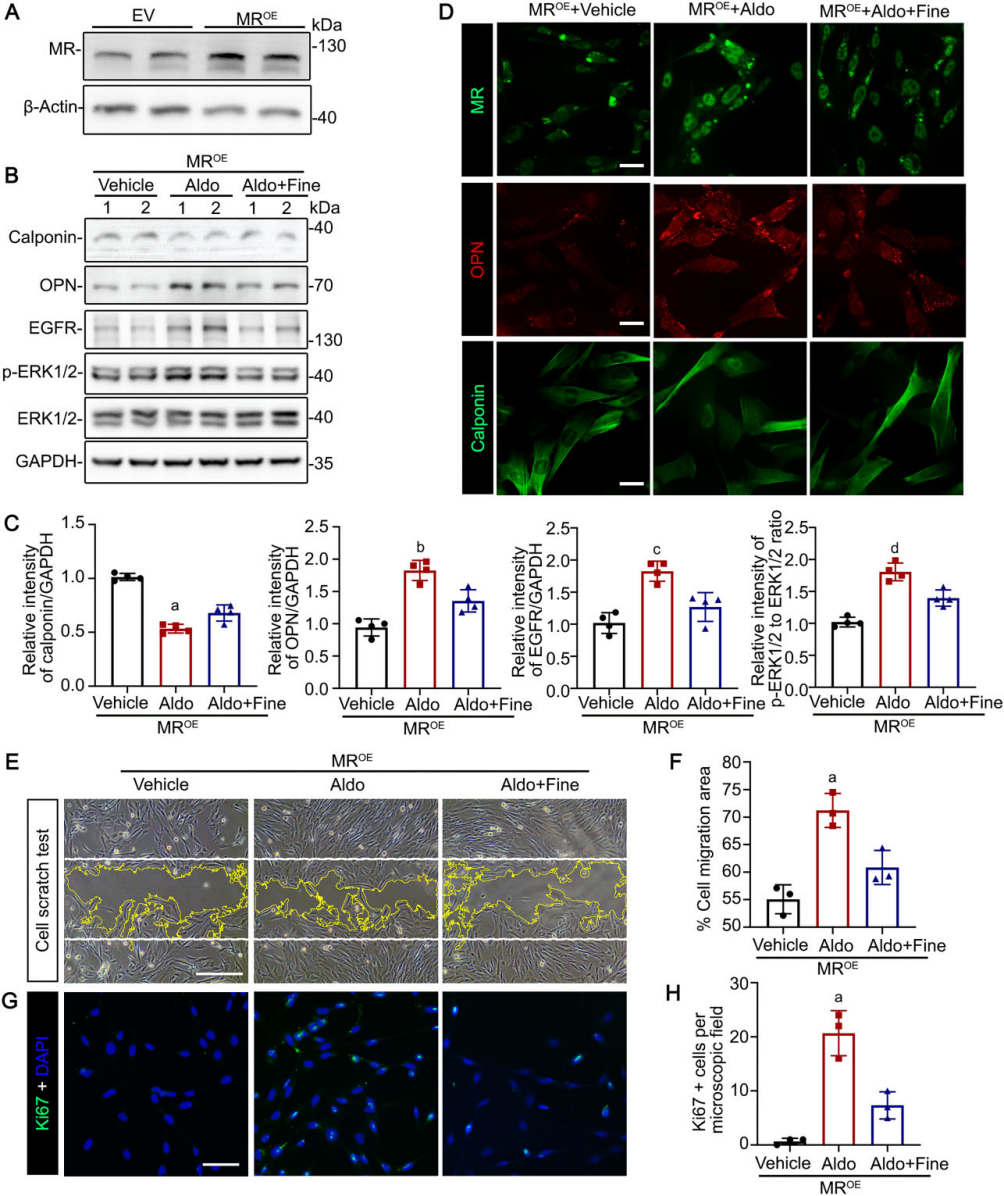
Mineralocorticoid receptor (MR) modulates VSMC phenotypic switching *in vitro*. VSMC were subjected to transfection with lentiviral vectors for MR overexpression, followed by being treated with aldosterone (Aldo, 100 nM) or finerenone (Fine, 40 nM) for 24 h. (**A**) VSMC transduced with empty vector (EV) or lentiviral vectors for MR overexpression (MR^OE^) were processed and subjected to immunoblot analysis for MR expression. (**B**) Cell lysates from different treatments were subjected to immunoblot analysis for calponin, osteopontin (OPN), EGFR, p-ERK1/2, ERK1/2 as well as GAPDH. (**C**) Quantification of the calponin, OPN, EGFR, p-ERK1/2, ERK1/2, and GAPDH expression levels by integrated density analyses of immunoblots, expressed as relative expression density normalized to GAPDH or ERK1/2. ^a^*P* = 0.0143, ^b^*P* = 0.0068, ^c^*P* = 0.0064, ^d^*P* = 0.0047 versus finerenone treatment group (n = 4). (**D**) Cells from different treatments were processed for immunofluorescent stained with MR, OPN, or calponin. Representative micrographs are shown. Scale bar = 10 µm. (**E**) Confluent VSMC monolayers were subjected to different treatments followed by being scraped with a 10 μL pipette. Phase-contrast micrographs were taken after indicated treatments for 12 h and processed for the invading front using ImageJ software. Scale bar = 50 µm. (**F**) Quantification by computerized morphometric analysis of the cell migration area. ^a^*P* = 0.0147 versus finerenone treatment group (n = 3). (**G**) VSMC with different treatments were processed for ki67 staining and counterstained with DAPI. Scale bar = 25 µm. (**H**) Absolute account of ki67-positive staining cells per microscopic field. ^a^*P* = 0.0090 versus finerenone treatment group (n = 3).

Previous data suggests the epidermal growth factor receptor-extracellular signal-regulated kinase 1/2 (EGFR-ERK1/2) pathway plays a pivotal role in the phenotypic switching of VSMC. Additionally, MR signaling has been implicated in the regulation of EGFR expression. To elucidate whether MR directly modulates VSMC phenotype, VSMC were subjected to forced expression of ectopic MR and treated with the MR agonist aldosterone in the presence or absence of the MR antagonist finerenone. Successful forced expression of MR was confirmed by immunoblot analysis of MR (Fig. 3A). As shown by immunoblot analysis or immunofluorescence (Fig. 3B-D), aldosterone elicited a synthetic phenotype in VSMC, characterized by *de novo* expression of OPN, along with decreased expression of calponin, a contractile marker of VSMC. Notably, while aldosterone treatment of non-transfected VSMC also triggered phenotypic switching, it was less effective in MR-overexpressed VSMC (Supplementary Fig. 1A-B). Additionally, MR activation by aldosterone led to an increased expression of EGFR and phosphorylation of ERK1/2, while treatment of finerenone mitigated the effects of aldosterone, restoring the contractile phenotype of VSMC. To investigate the influence of MR activation on VSMC migration, the confluent monolayer of VSMC was mechanically scraped with a 10 μl pipette tip, followed by different treatments. Migration was quantified by measuring the area between the invading front at 12 hours post-injury. As shown in Fig. 3E&F, MR activation by aldosterone significantly enhanced VSMC motility, as evidenced by an increase in the percentage of migration area. Notably, co-treatment with finerenone effectively attenuated this migratory response, indicating a regulatory role for MR in VSMC migration. In addition, cellular proliferation was significantly increased by aldosterone treatment. However, this effect was notably mitigated by co-treatment with finerenone (Fig. 3G&H).

**Figure 4:**
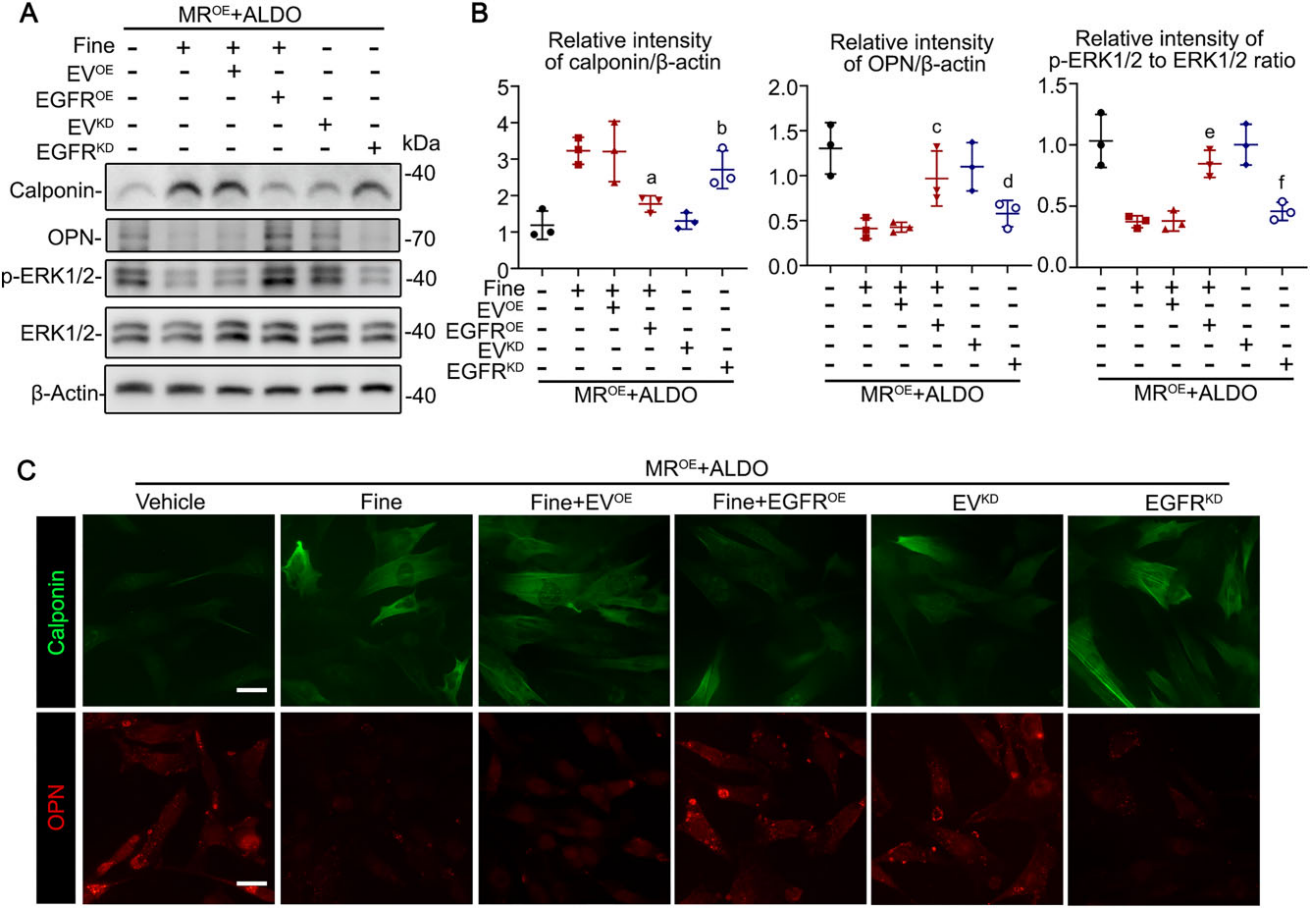
Mineralocorticoid receptor (MR) modulates VSMC phenotypic switching *via* the EGFR-ERK1/2 pathway. (**A**) Cell lysates from various treatments underwent immunoblot analysis for calponin, osteopontin (OPN), p-ERK1/2, ERK1/2 as well as β-actin. (**B**) Quantification of the calponin, OPN, p-ERK1/2, ERK1/2, and β-actin expression levels by integrated density analyses of immunoblots, expressed as relative expression density normalized to β-actin or ERK1/2. ^a^*P* = 0.0439, ^c^*P* = 0.0398, ^e^*P* = 0.0042 versus MR^OE^ + Aldo + Fine + EV^OE^ groups (n = 3). ^b^*P* = 0.0126, ^d^*P* = 0.0420, ^f^*P* = 0.0067 versus MR^OE^ + Aldo + EV^KD^ groups (n = 3). (**C**) Cells from various treatments were subjected to immunofluorescent stained with OPN, or calponin. Representative micrographs are shown. Scale bar = 10 µm.

To test if EGFR mediates the phenotypic modulation of MR signaling, VSMC were forced expression of EGFR or EGFR siRNA in MR-overexpressing VSMC, followed by treatment with aldosterone. As shown in Fig. 4, EGFR knockdown mimicked the protective effect of finerenone on VSMC. Conversely, overexpression of EGFR diminished the effect of finerenone, associated with enhanced phosphorylation of ERK1/2, as evidenced by immunoblot analysis (Fig. 4A&B) or immunofluorescent staining (Fig. 4C). These results suggested MR regulating VSMC phenotypic switching *via*, at least in part, the EGFR-ERK1/2 pathway.

### MR antagonist improves fistula function in a rat AVF model

**Figure 5:**
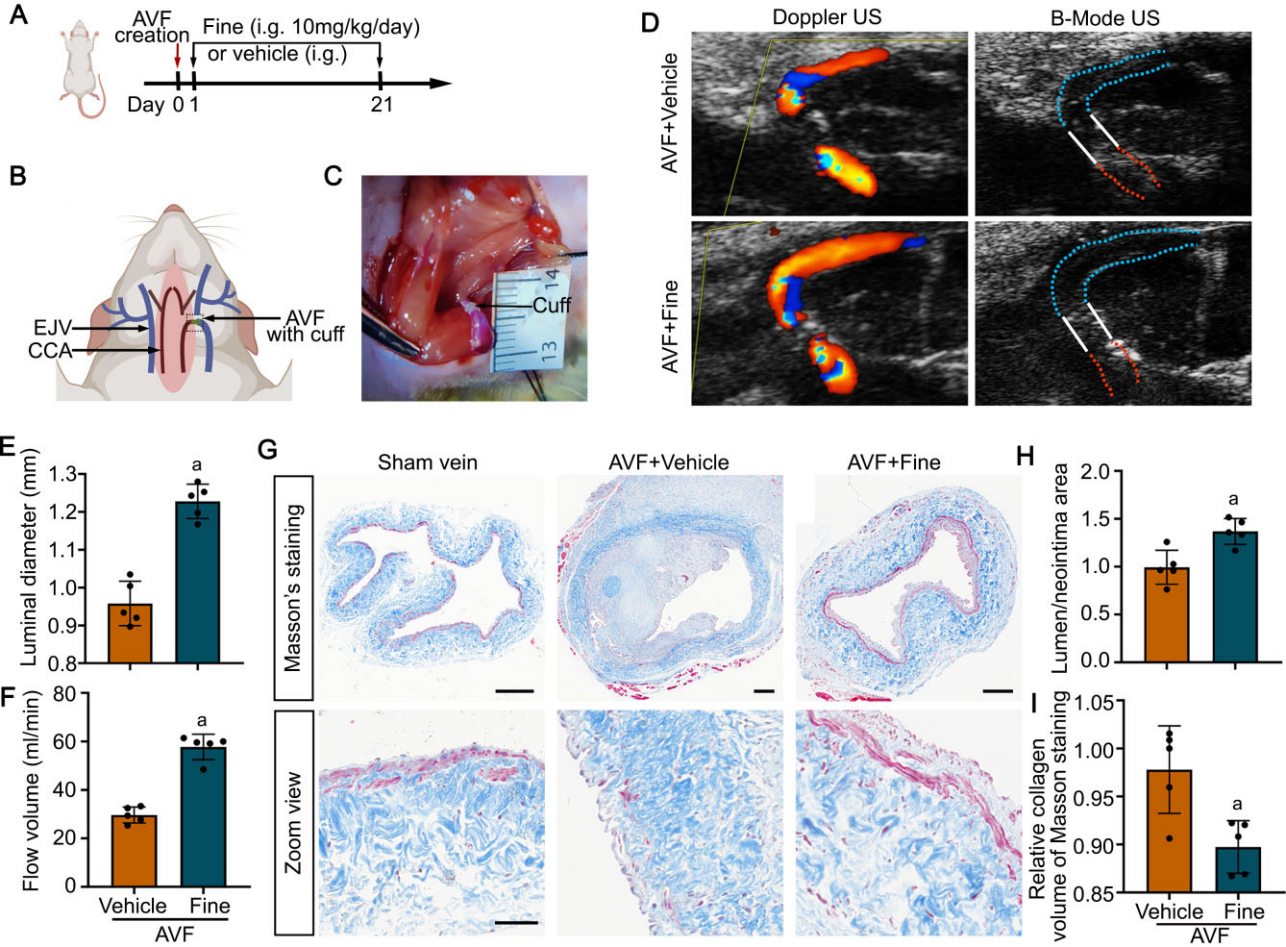
Mineralocorticoid receptor (MR) antagonist improved vascular fibrosis and neointima hyperplasia in a rat arteriovenous fistula (AVF) model. (**A**) The schematic diagram illustrates animal experimental design. (**B**) Schematic diagram depicting AVF creation in rats. (**C**) Representative views of the AVF anastomosis with cuff. (**D**) AVF was evaluated around the anastomosis by ultrasound (US). (**E**) AVF luminal diameters were measured at the outflow vein, 2 mm from the distal end of the cuff. ^a^*P* < 0.0001 versus AVF + vehicle group (n = 5). (**F**) Flow volume was measured. ^a^*P* < 0.0001 versus AVF + vehicle group (n = 5). (**G**) The outflow vein sections were processed for Masson's trichrome staining. Scale bar = 50 µm. (**H**) Quantification by computerized morphometric analysis of lumen/neointima area for AVF with or without finerenone treatment. ^a^*P* = 0.0056 versus AVF + vehicle group (n = 5). (**I**) Quantification of relative collagen volume in media of Masson's trichrome staining. ^a^*P* = 0.0095 versus AVF + vehicle group (n = 5). Fine, finerenone.

A plethora of data demonstrated that the nonsteroidal MR antagonist finerenone exerts protective effects against cardiovascular injury. To examine whether finerenone has beneficial effects on vascular remodeling in AVF, we established a rat AVF model by connecting the common carotid artery with the external jugular vein using a cuff. Subsequently, some rats with AVF were treated with finerenone (Fig. 5A-C). As shown in Fig. 5D-F, finerenone significantly increased both the luminal diameter and the flow volume of the outflow vein in rat AVF, as assessed by ultrasonography. Moreover, finerenone treatment notably mitigated neointima hyperplasia and venous fibrosis, as confirmed by Masson's trichrome staining and computerized analysis of lumen/neointima area and relative collagen volume in rat AVF (Fig. 5G-I).

### MR antagonist modulates VSMC phenotypic switching in a rat AVF model

**Figure 6:**
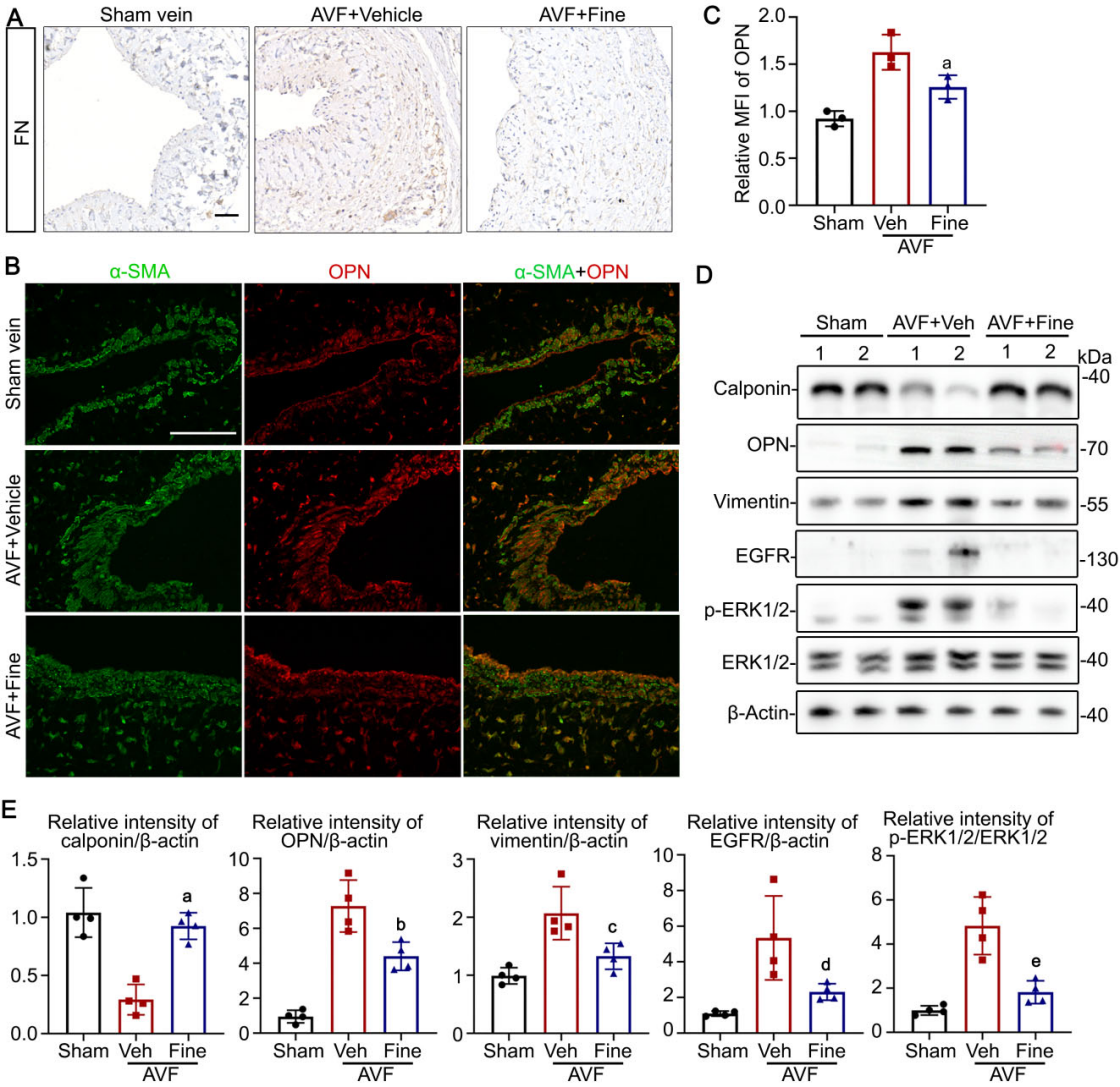
Mineralocorticoid receptor (MR) antagonist attenuated VSMC phenotypic switching. (**A-B**) The sections of AVF outflow vein were processed for immunohistochemistry staining of fibronectin (FN) or immunofluorescent staining of smooth muscle α-actin (α-SMA) with osteopontin (OPN). Scale bar = 50 µm. (**C**) Quantification of mean fluorescence intensity (MFI) of OPN. ^a^*P* = 0.0460 versus AVF + vehicle group (n = 3). (**D**) Immunoblot analysis of AVF specimens for calponin, OPN, vimentin, EGFR, p-ERK1/2, ERK1/2, and β-actin. (**E**) Quantification of the calponin, OPN, vimentin, EGFR, p-ERK1/2, ERK1/2, and β-actin expression levels by integrated density analyses of immunoblots, expressed as relative expression density normalized to β-actin or ERK1/2. ^a^*P* = 0.0003, ^b^*P* = 0.0146, ^c^*P* = 0.0266, ^d^*P* = 0.0450, ^e^*P* = 0.0052 versus AVF + vehicle group (n = 4).

The VSMC phenotype in a rat AVF model was evaluated. In a rat AVF model, VSMC was switched from a contractile phenotype to a synthetic phenotype, characterized by diminished expression of calponin, alongside elevated expression of OPN, fibronectin, and vimentin, as manifested by immunoblotting or immunohistochemistry staining (Fig. 6A-E). Notably, these phenotypic changes were ameliorated following treatment with finerenone. Consistent with the *in vitro* findings, finerenone treatment also suppressed the activated EGFR-ERK1/2 pathway, marked by reduced expression levels of EGFR and p-ERK1/2, as shown by immunoblot analysis (Fig. 6D&E).

### MR antagonist improves nitric oxide productivity in endothelial cells

Finerenone treatment on rat AVF has a significant protective effect on neointima hyperplasia and venous fibrosis, both of which may be the results of VSMC phenotypic switching. Additionally, MR activity in endothelial cells (ECs) is crucial for vascular injury response [19]. Specifically, MR in ECs has been shown to influence nitric oxide (NO) production, a key factor associated with vascular function. To decipher whether finerenone improves rat AVF blood flow and luminal diameter by elevated NO productivity in ECs, EC was subjected to forced expression of ectopic MR and treated with the MR agonist aldosterone in the presence or absence of the MR antagonist finerenone. As shown by H2DCF DA and DAF-FM DA fluorescent staining, which represents the cell rective oxygen species (ROS) or NO levels, respectively (Supplementary Fig. 2A-D), aldosterone markedly increased ROS level while decreasing NO level in ECs, particularly in MR-overexpressing cells. In contrast, these effects were markedly attenuated by finerenone treatment. Furthermore, MR activation by aldosterone led to a reduction in phosphorylation of eNOS at Ser1177 (Supplementary Fig. 2E-F), which is critical for NO production. This effect was reversed by finerenone, indicating that the protective effects of finerenone on AVF function were not only attributed to modulation of VSMC phenotype, but also mediated through its ability to regulate endothelial function and restore balance in NO signaling.

## DISCUSSION

**Figure 7:**
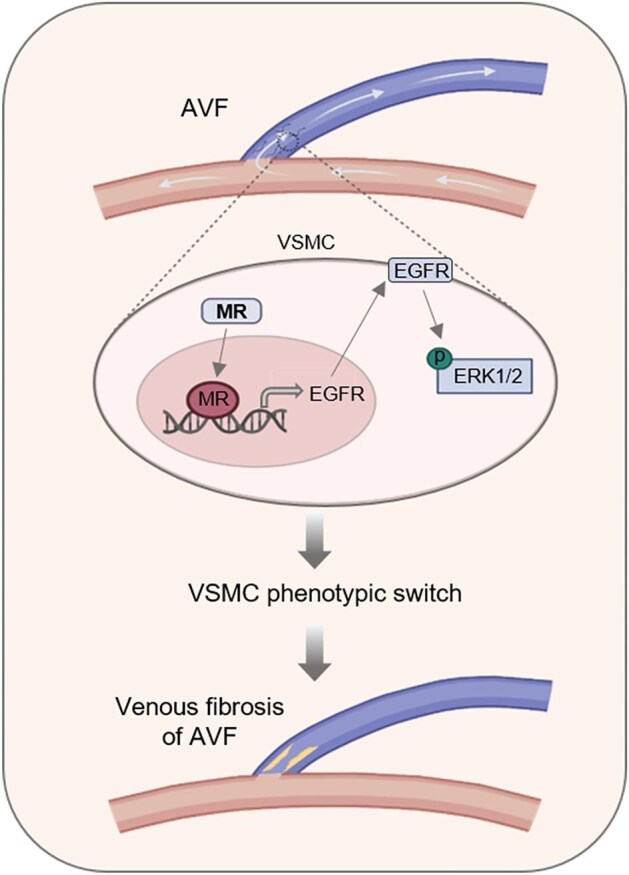
Schematic depicts mineralocorticoid receptor (MR) overexpression driving arteriovenous fistula (AVF) dysfunction by modulating VSMC phenotype switching. In the juxta-anastomotic region of AVF, hemodynamic shear stress triggers MR overactivation in VSMC. Subsequently, the overactivation of MR leads to increased expression of EGFR, followed by heightened phosphorylation of ERK1/2, facilitating the phenotypic switching of VSMC and ultimately resulting in AVF dysfunction. Therefore, this detrimental process could be retarded by MR antagonists.

AVF, the Achilles’ heel of hemodialysis therapy, is plagued by a notably high failure rate attributed to venous stenosis [20]. However, despite various attempts, there are still no effective ways to improve the long-term patency of fistula in clinical practice. Hence, there is a pressing need for a more comprehensive pathophysiology of AVF dysfunction and the development of novel therapeutic targets for AVF dysfunction. Here, in this study, we present novel insights indicating the overactivation of MR in dysfunctional AVF, which promotes VSMC phenotypic transition towards a synthetic state *via* the EGFR-ERK1/2 pathway, ultimately contributing to fistula stenosis. Moreover, our findings suggest that the MR antagonist may hold promise in retarding the progression of fistula stenosis in hemodialysis patients (Fig. 7).

As a member of the steroid receptor family, MR plays a multifaceted role, not only in blood pressure regulation but also in modulating vascular-specific gene expression programs that govern vascular function. Mounting evidence suggests that MR activation contributes to adverse vascular remodeling by instigating vascular inflammation, stiffness, and oxidative stress [21]. For instance, in patients with primary aldosteronism, increased vascular intima-media thickness and fibrotic, stiffened vasculature have been observed [11], and the plasma aldosterone levels were independent predictors of in-stent restenosis [22]. Animal models have further elucidated the detrimental effects of MR activation, low-dose aldosterone infusion significantly exacerbated the wire-induced vascular injury, characterized by increased ECM deposition and vascular medial area [23]. Moreover, the activation of MR was significantly increased in human vein bypass graft [24] and here in dysfunctional AVF. Additionally, conditional overexpression of MR in mouse vascular ECs has been associated with increased contractile response of mesenteric arteries [25]. Consistent with these findings, our study unveils MR overactivation in dysfunctional AVF. Furthermore, we provide compelling evidence that MR overactivation in cultured VSMC mediates a phenotypic switching from a contractile to a synthetic state in response to aldosterone stimulation.

The salutary effects of MR antagonists have been proven in cardiovascular disease, even in patients with chronic kidney disease [26, 27], by promoting cardiovascular remodeling in clinical investigations. Recently, emerging evidence revealed MR antagonists also exerted a beneficial effect on vascular injury by attenuating intimal hyperplasia and VSMC dysfunction. For instance, the highly selective MR antagonist eplerenone demonstrated the ability to attenuate neointimal hyperplasia in a swine model with coronary stent implantation [28]. Additionally, the nonsteroidal MR antagonist finerenone effectively mitigated neointimal lesion formation following wire-induced artery injury by reducing VSMC proliferation and EC apoptosis. Furthermore, vein graft remodeling was also improved by the classical MR antagonist spironolactone in a mouse model [13]. Likewise, in the present study, finerenone prevented cell phenotypic switching from contractile phenotype to synthetic phenotype in aldosterone induced-VSMC injury. The study further revealed that finerenone treatment was correlated with the patency of AVF in a rat AVF model, indicating MR antagonists may hold the promise of improving fistula stenosis in hemodialysis patients. However, the current clinical application of MR antagonists in hemodialysis patients with kidney failure remains extremely limited due to the risk of hyperkalemia. Nevertheless, a growing body of evidence suggests that the treatment of MR antagonists may improve clinical outcomes in dialysis patients without a significant increase in the risk of hyperkalemia [26, 29–32]. The underlying reason may be that the majority of the potassium is eliminated by hemodialysis rather than in urine [29]. Therefore, MR antagonists still hold promise to be applied in hemodialysis patients to improve cardiac outcome and AVF function.

How does MR mediate AVF vascular remodeling? Our study highlighted the central role of VSMC phenotypic switching, which accounts for the pathogenesis of vascular diseases, including atherosclerosis, aneurysm, and AVF [33–36]. In healthy conditions, VSMC exhibit a differentiated quiescent contractile state and rarely proliferate. However, VSMC may dedifferentiate to a variety of distinct phenotypes under stimuli. Notably, synthetic VSMC accounts for the neointima hyperplasia and ECM deposition in the vasculature, contributing to vascular fibrosis. Previous research has identified several mechanisms of MR-mediated VSMC phenotypic switching [37, 38]. For instance, aldosterone-induced OPN expression and VSMC migration in rat VSMC in an MR-dependent manner *in vitro* [39, 40]. Besides, *in vivo* study, the MR signaling was also indicated to mediate VSMC proliferation, ECM deposition, and medial vessel thickening in a wire injury mouse model [23, 14]. Consistently, our study demonstrated that MR modulated VSMC phenotype involving the EGFR-ERK1/2 pathway. MR may mediate EGFR transactivation through non-genomic functions [41] and induce EGFR expression through genomic action [42]. Subsequently, cytosolic signaling cascades are activated, like ERK1/2, which has been shown to regulate VSMC function [43].

In addition to the effect of MR on VSMC phenotypic switching, the MR in ECs also plays a crucial role in various vascular remodeling. Numerous studies have shown that MR in endothelial cells plays a functional role in NO regulation. For instance, studies have shown that the aldosterone-MR signaling pathway in ECs promotes production of superoxide and ROS, contributing to vascular oxidative stress [44, 45]. Conversely, MR antagonists have been shown to restore NO production and bioavailability by attenuating eNOS uncoupling in humans and animals [46]. Consistent with these findings, our study revealed that finerenone enhanced NO production by attenuating aldosterone-MR-induced reductions eNOS phosphorylation at ser177. This study suggests that the protective effects of finerenone on rat AVF function were not only attributed to modulation of VSMC phenotype, but also mediated through its ability to regulate endothelial function and restore balance in NO signaling.

A key limitation of this study is the choice of control tissue. Ideally, a non-dysfunctional AVF would have been used as control to the dysfunctional AVF samples. However, due to the irrationality of procuring non-dysfunctional AVF tissues in hemodialysis patients, the current studies [47, 48] and this study employed normal veins as controls. While this allowed us to examine baseline differences in vascular structure and function, the use of normal veins may not fully capture the complexity of AVF pathophysiology. As a result, the direct comparison between normal veins and dysfunctional AVFs may limit the ability to discern the specific molecular and cellular changes associated solely with AVF dysfunction.

In summary, MR activation in VSMC contributed to AVF dysfunction by modulating VSMC phenotypic switching. MR antagonist treatment improved the outcomes of AVF in rat models. Mechanistically, MR mediated VSMC phenotypic switching involving the EGFR-ERK1/2 pathway. Our findings indicate MR antagonism may be a promising therapy for retarding the progression of fistula stenosis in patients on hemodialysis.

## Supplementary Material

gfae247_Supplemental_File

## Data Availability

The authors agree the data supporting the findings of this study are available upon request.
