# Supplementary material for: Mineralocorticoid receptor antagonism attenuates arteriovenous fistula stenosis by modulating the phenotype of vascular smooth muscle cells
Source: Nephrol Dial Transplant. 2024 Nov 7;40(6):1124–36. doi: 10.1093/ndt/gfae247 (PMC12123322; doi:10.1093/ndt/gfae247)
Supplement: gfae247_Supplemental_File [file gfae247_supplemental_file.pdf]

Supplemental materials

Mineralocorticoid receptor antagonism attenuates arteriovenous fistula stenosis by modulating the phenotype of vascular smooth muscle cells

Yamin Liu[1,2,#], Bohan Chen[1,3,#], Kai Chen[4], Yufei Wang[1], Chunyu Zhou[1], Xianhui Liang[1,3,*], Kai Wang[1,2,*], Pei Wang[1,3,*]

[1]Blood Purification Center, Department of Nephrology, the First Affiliated Hospital of Zhengzhou University, Zhengzhou, Henan, China; [2]Key Laboratory of Bioactive Materials, Ministry of Education, College of Life Science, Nankai University, Tianjin, China; [3]Research Institute of Nephrology, Zhengzhou University, Zhengzhou, China; [4]Department of Nephrology, Kaifeng People's Hospital, Kaifeng, China.

[#]Yamin Liu and Bohan Chen are co-first authors that contributed equally to this work.

*Correspondence: Pei Wang, Email: wpei@zzu.edu.cn; Kai Wang, Email: 013053@nankai.edu.cn; and Xianhui Liang, Email: fccliangxh@zzu.edu.cn.

Cell culture

Human primary human umbilical artery endothelial cells were obtained from Aoyinbio Co. (Shanghai, China) and was grown in endothelial cell medium (ScienCell Research Laboratories, USA) at 37 ℃ [16]. Cells were transfected with lentivirus coding for human NR3C2 cDNA (GENECHEM, China) according to the manufacturer's instructions. After stable transfection, cells were treated with aldosterone (100 nM, Sigma-Aldrich, Germany) or finerenone (40 nM, Sigma-Aldrich) treatment for 24 h. After the indicated treatment, cells were processed for further examination.

Human umbilical artery VSMC was obtained from Aoyinbio Co. (Shanghai, China) and was grown in Dulbecco's modified Eagle's medium/F12 containing 10% FBS at 37 ℃ [16]. Cells were transfected with lentivirus coding for human NR3C2 cDNA (GENECHEM, China) according to the manufacturer's instructions. After stable transfection, cells were treated with aldosterone (100 nM, Sigma-Aldrich, Germany) or finerenone (40 nM, Sigma-Aldrich) treatment for 24 h. After the indicated treatment, cells were processed for further examination.

Detection of reactive oxygen species (ROS) and nitric oxide (NO) production

Generation of ROS and NO was assessed using the H2DCFDA Assay Kit (MedChemExpress, China) and DAF-FM DA kit (Beyotime, China), respectively. Briefly, cells were exposed to various treatments and then ROS detection reagent or NO detection reagent was added for 10~20 min according to the manufacturer's instructions. Fluorescent images were acquired using a Nikon fluorescence microscope.

Supplemental figures

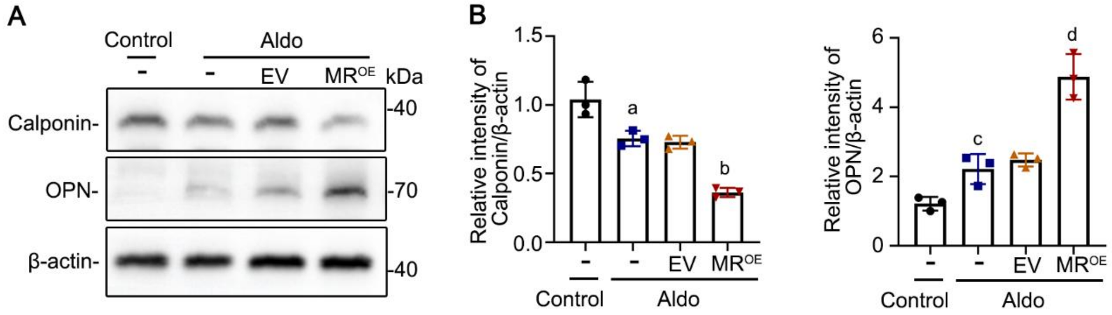

Supplemental Figure 1: Aldosterone induced vascular smooth muscle cell (VSMC) phenotypic switching *in vitro*. VSMC were subjected to transfection with lentiviral vectors for MR overexpression, followed by being treated with aldosterone (Aldo, 100 nM) for 24 hours. (A) Cell lysates from different treatments were subjected to immunoblot analysis for calponin, osteopontin (OPN) as well as β-actin. (B) Quantification of the calponin, OPN and β-actin expression levels by integrated density analyses of immunoblots, expressed as relative expression density normalized to β-actin. ^{a}P=0.0259, ^{c}P=0.0219 versus control group. ^{b}P=0.0004, ^{d}P=0.0037 versus EV+Aldo treatment group (n=3).

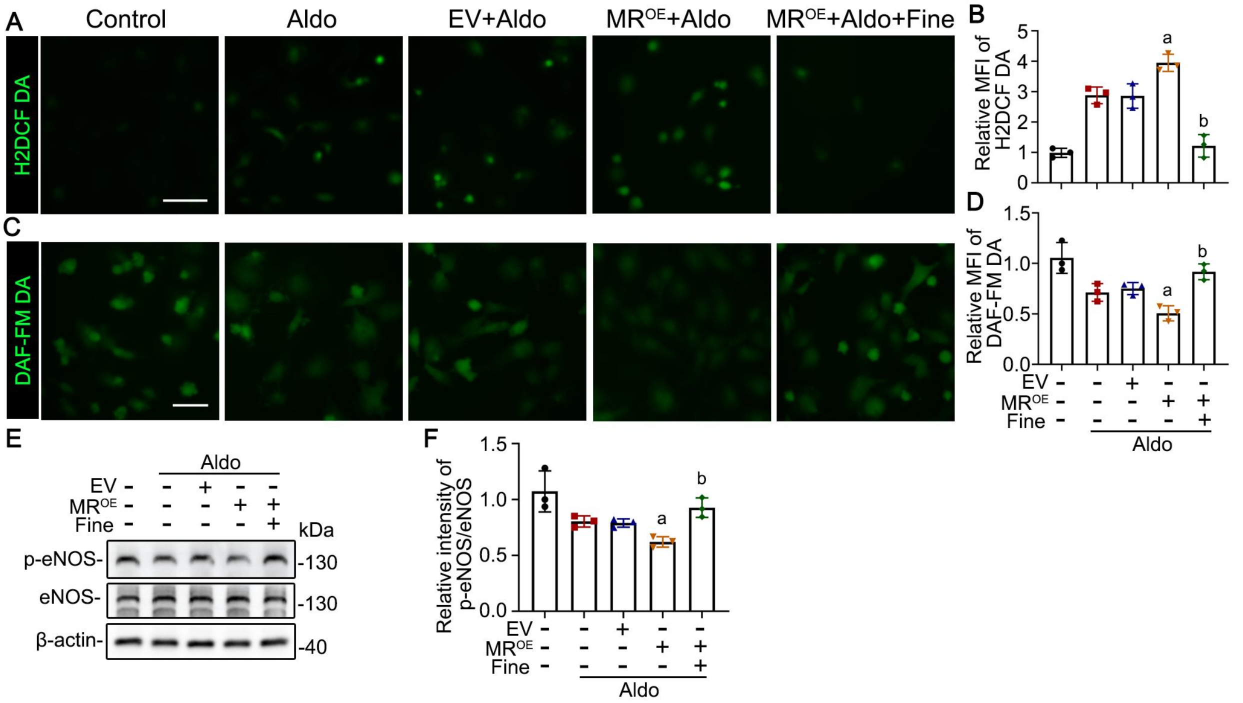

Supplemental Figure 2: Mineralocorticoid receptor (MR) antagoist increased nitric oxide (NO) productivity in endothelial cells. Endothelial cells were subjected to transfection with lentiviral vectors for MR overexpression, followed by being treated with aldosterone (Aldo, 100 nM) for 24 hours or finerenone (Fine, 40 nM) for 24 hours. (A) Assessment of cellular ROS levels by detecting H2DCF DA. (B) Quantification of mean fluorescence intensity (MFI) of H2DCF DA. ^{a}P=0.0005 versus EV+Aldo treatment group. ^{b}P=0.0183 versus MR^{OE}+Aldo treatment group (n=3). (C) Assessment of cellular NO levels by detecting DAF-FM DA. (D) Quantification of mean fluorescence intensity (MFI) of DAF-FM DA. ^{a}P=0.0027 versus EV+Aldo treatment group. ^{b}P=0.0110 versus MR^{OE}+Aldo treatment group (n=3). (E) Cell lysates from different treatments were subjected to immunoblot analysis for p-eNOS, eNOS as well as β-actin. (F) Quantification of the p-eNOS and eNOS expression levels by integrated density analyses of immunoblots, expressed as relative expression density normalized to eNOS. ^{a}P=0.0083 versus control group. ^{b}P=0.0058 versus EV+Aldo treatment group (n=3).
